# *Mycobacterium tuberculosis* secretory proteins downregulate T cell activation by interfering with proximal and downstream T cell signalling events

**DOI:** 10.1186/s12865-015-0128-6

**Published:** 2015-11-09

**Authors:** Bhawna Sharma, Rajni Upadhyay, Bhavyata Dua, Naim Akhtar Khan, Vishwa Mohan Katoch, Bharat Bajaj, Beenu Joshi

**Affiliations:** Department of Immunology, National JALMA Institute for Leprosy and Other Mycobacterial Diseases (ICMR), Dr.M.Miyazaki Marg, Tajganj, Agra, 282001 India; UPRES EA 4183 Lipides & Signalisation Cellulaire, Faculté des Sciences de la vie, Université de Bourgogne, 6, Boulevard Gabriel, Dijon, 21000 France; Formerly in Department of Health Research and ICMR, Ansari Nagar, New Delhi-29 India; State TB Training & Demonstration Centre, S.N. Medical College Campus, Agra, 282 002 India

## Abstract

**Background:**

*Mycobacterium tuberculosis* (*M. tuberculosis*) modulates host immune response, mainly T cell responses for its own survival leading to disease or latent infection. The molecules and mechanisms utilized to accomplish immune subversion by *M. tuberculosis* are not fully understood. Understanding the molecular mechanism of T cell response to *M. tuberculosis* is important for development of efficacious vaccine against TB.

**Methods:**

Here, we investigated effect of *M. tuberculosis* antigens Ag85A and ESAT-6 on T cell signalling events in CD3/CD28 induced Peripheral blood mononuclear cells (PBMCs) of PPD+ve healthy individuals and pulmonary TB patients. We studied CD3 induced intracellular calcium mobilization in PBMCs of healthy individuals and TB patients by spectrofluorimetry, CD3 and CD28 induced activation of mitogen activated protein kinases (MAPKs) in PBMCs of healthy individuals and TB patients by western blotting and binding of transcription factors NFAT and NFκB by Electrophorectic mobility shift assay (EMSA).

**Results:**

We observed CD3 triggered modulations in free intracellular calcium concentrations in PPD+ve healthy individuals and pulmonary TB patients after the treatment of *M. tuberculosis* antigens. As regards the downstream signalling events, phosphorylation of MAPKs, Extracellular signal-regulated kinase 1 and 2 (ERK1/2) and p38 was curtailed by *M. tuberculosis* antigens in TB patients whereas, in PPD+ve healthy individuals only ERK1/2 phosphorylation was inhibited. Besides, the terminal signalling events like binding of transcription factors NFAT and NFκB was also altered by *M. tuberculosis* antigens. Altogether, our results suggest that *M. tuberculosis* antigens, specifically ESAT-6, interfere with TCR/CD28-induced upstream as well as downstream signalling events which might be responsible for defective IL-2 production which further contributed in T-cell unresponsiveness, implicated in the progression of disease.

**Conclusion:**

To the best of our knowledge, this is the first study to investigate effect of Ag85A and ESAT-6 on TCR- and TCR/CD28- induced upstream and downstream signalling events of T-cell activation in TB patients. This study showed the effect of secretory antigens of *M. tuberculosis* in the modulation of T cell signalling pathways. This inflection is accomplished by altering the proximal and distal events of signalling cascade which could be involved in T-cell dysfunctioning during the progression of the disease.

**Electronic supplementary material:**

The online version of this article (doi:10.1186/s12865-015-0128-6) contains supplementary material, which is available to authorized users.

## Background

Tuberculosis (TB) is a major global health problem and still remains one of the world’s deadliest communicable diseases. In 2013, an estimated 9.0 million people developed TB and 1.5 million died from the disease, 360 000 of whom were HIV-positive [1]. The resurgence of TB worldwide has intensified research efforts directed on examining the host defence and pathogenic mechanisms operative in *Mycobacterium tuberculosis* (*M. tuberculosis*) infection. This is primarily because the organism lives inside the cells and thus T cells, rather than antibodies are required to eliminate bacteria [2, 3]. For proper T-cell activation, T cell receptor (TCR) engagement with antigen presenting cells (APCs) in presence of co-stimulation is required and any variation in this, could lead to T-cell anergy [4, 5]. The pathogenic mycobacteria resides inside macrophage and involves in the inhibition of several host-cell procedures, which allows its survival inside the host cells. The host processes that are hampered by pathogenic bacteria, the molecules and mechanisms utilized by pathogenic mycobacteria to accomplish intracellular survival are not fully understood. *M. tuberculosis* can modulate the adaptive immune response using various mechanisms directed on both the APCs and the T cells. *M. tuberculosis* infected macrophages indirectly suppress T cell activation by interfering with antigen processing and presentation. Seitzer et al. showed decreased expression of CD3-ζ, a key signalling domain of the TCR/CD3 complex in T cells from human TB patients [6]. Wang et al. have shown that the potent T cell antigen ESAT-6 can directly suppress IFN-γ production in CD4+ T cells [7]. Directly inhibition of polyclonal murine CD4+ T cell activation by *M. tuberculosis* cell wall glycolipids by blocking ZAP-70 phosphorylation has been shown by Mahon et al. [8] and later they extended their study by reporting ManLAM induced inhibition of TCR signalling by interference with ZAP-70, Lck and LAT phosphorylation in antigen-specific murine CD4+ T cells and primary human T cells [9]. Recently regulation of IFN-γ production by ERK and p38 MAPK signalling pathway and through SLAM costimulation has been suggested in TB [10].

Secretory antigens of *M. tuberculosis* are immunodominant and could have a role in the outcome of disease by modulation of cell signalling pathways*.* Inhibition of IFN-γ production through p38 MAPK pathway by ESAT-6 has been reported in T cells from healthy individuals [11]. However, mechanisms of action of *M. tuberculosis* antigens (specifically Ag85A and ESAT-6) on TCR/CD28 mediated signalling in TB patients have not been addressed till date hence needs to be investigated. In the present study effect of Ag85A, ESAT-6 (secretory antigens), Purified protein derivative (PPD) (a common antigen) and H_37_Rv (laboratory strain commonly used) was investigated on TCR/CD28-triggered signalling which could be involved in T-cell dysfunctioning that leads to bacterial survival hence disease progression. Our objective was to study calcium mobilization, activation of MAPKs and binding of NFAT and NFκB on IL-2 promoter in peripheral blood mononuclear cells (PBMCs) of pulmonary TB patients and healthy individuals in presence or absence of *M. tuberculosis* antigens.

## Results

The concentration of antigens showing optimum proliferative index in lymphocyte transformation test (LTT) in PBMCs of pulmonary TB patients and PPD+ve healthy individuals (data not shown) was considered as optimal working concentration for further signalling experiments. Standardized doses (5 μg/ml for Ag85A and H_37_Rv and 10 μg/ml for ESAT-6) showed stimulation indices (S.I.) more than two in PPD + ve healthy individuals as well as in pulmonary TB patients. The levels of S.I. with antigens (5 μg/ml for Ag85A and H_37_Rv and 10 μg/ml for ESAT-6) were significantly lower in TB patients than PPD+ve healthy individuals and were also significantly lower to the S.I. of PHA and PPD stimulated cells (Additional file 1: Figure S1A-C). Serial concentrations of antigens (2.5 μg/ml, 5 μg/ml, 7.5 μg/ml, 10 μg/ml and 15 μg/ml) were used in LTT and optimal dose of antigen found by LTT results was observed to show the maximum effect.

### *M. tuberculosis* antigens differentially curtail TCR triggered intracellular calcium mobilization

**Fig. 1:**
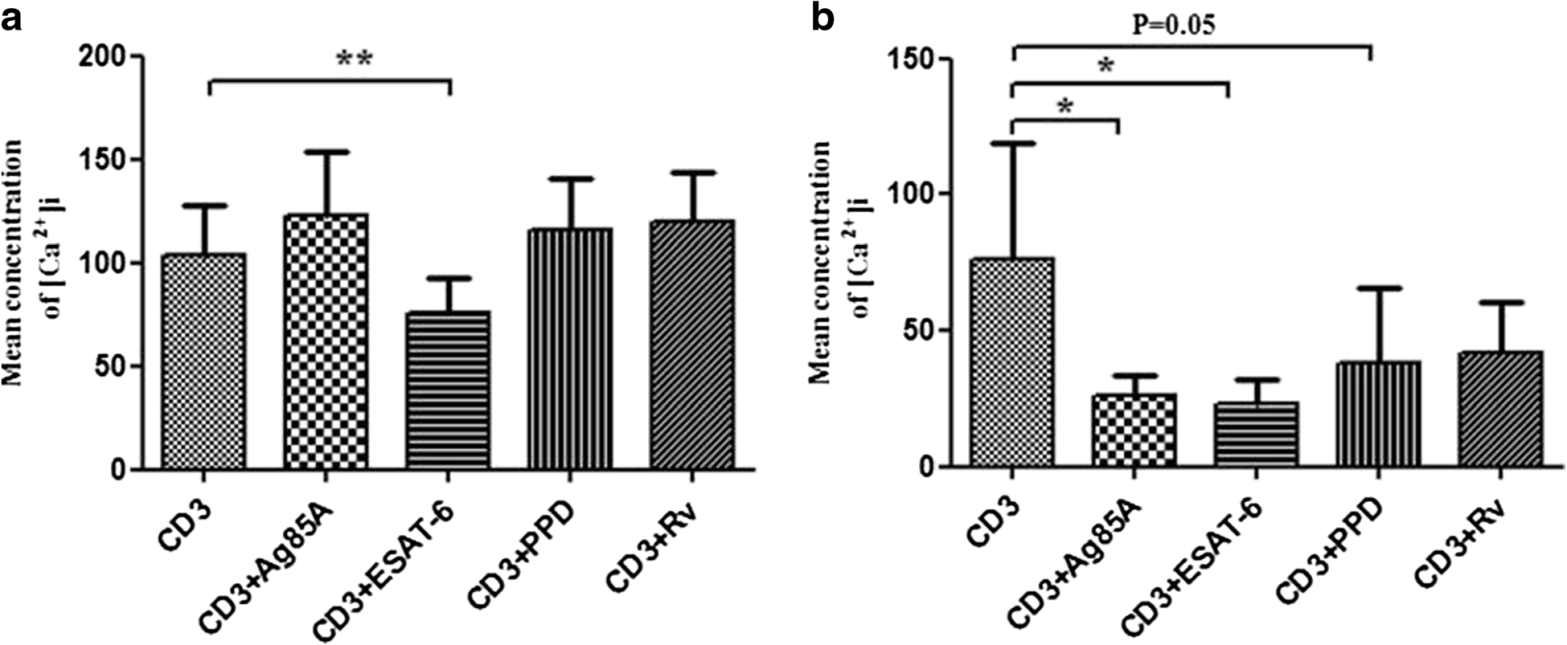
*M. tuberculosis* antigens alter free intracellular calcium concentration in PPD+ve healthy individuals and pulmonary TB patients. Peripheral blood mononuclear cells (PBMCs) from 13 PPD+ve healthy donors and five pulmonary TB patients were stimulated with *M. tuberculosis* antigens for 4 h. Fluorescence intensities were measured in ratio mode using Varian ECLIPSE spectrofluorometer as mentioned in material and methods. Bar diagrams show changes in [Ca2+]i in anti CD_3_ and ionomycin treated cells of (**a**) PPD+ve healthy individuals (**b**) Pulmonary TB patients and values in histogram are mean ± SEM. *, *P* < 0.05; **, *P* <0.005

To find out the effect of *M. tuberculosis* antigens on intracellular calcium mobilization, we measured intracellular calcium concentration by spectrofluorometer. We assessed TCR-triggered calcium mobilization by adding anti-CD3 antibody on cells pretreated with optimum doses of *M. tuberculosis* antigens (Ag85A, ESAT-6, PPD and H_37_Rv). We noticed that TCR triggered calcium mobilization in PPD+ve healthy individuals was significantly diminished by ESAT-6, but it was increased by Ag85A, PPD and H_37_Rv though it was not statistically significant. It showed that ESAT-6 inhibited the transmembrane calcium mobilization, while it was enhanced by other antigens. TCR triggered transmembrane calcium mobilization was significantly curtailed in pulmonary TB patients, by Ag85A and ESAT-6. PPD and H_37_Rv also inhibited the mobilization but it was not statistically significant (Fig. 1).

### *M. tuberculosis* antigens modulate TCR/CD28-induced MAPK activation

**Fig. 2:**
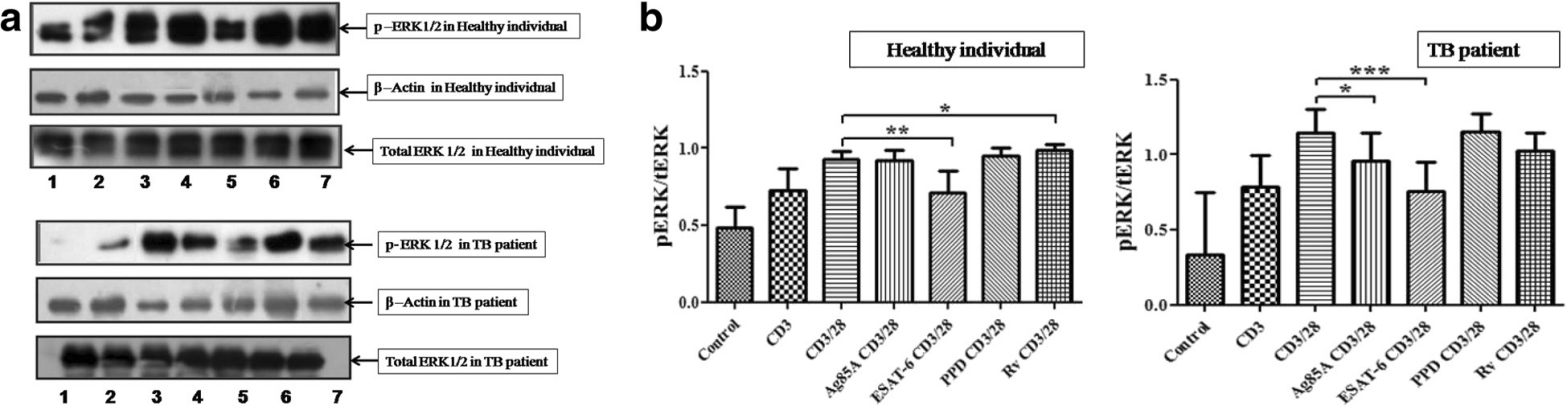
*M. tuberculosis* antigens modulate TCR- and TCR/CD28-induced phosphorylation of ERK1/2 in PPD+ve healthy individuals and pulmonary TB patients. (**a**) Peripheral blood mononuclear cells (PBMCs) from six PPD+ve healthy donors and 12 pulmonary TB patients were stimulated with *M. tuberculosis* antigens for 4 h. Cells were then activated with anti CD3 and anti CD28 antibodies (as mentioned in methodology). Phosphorylated ERK ½, total ERK (p- ERK ½ and tERK, respectively) and β- Actin expression were determined by western blot. One representative blot of healthy individual and TB patient is shown, where Lane 1- Control, Lane 2- anti CD3 stimulated, Lane 3- anti CD3 + anti CD28 stimulated, Lane 4- anti CD3 + anti CD28 stimulated in presence of Ag85A, Lane 5- anti CD3 + anti CD28 stimulated in presence of ESAT-6, Lane 6- anti CD3 + anti CD28 stimulated in presence of PPD and Lane 7-anti CD3 + anti CD28 stimulated in presence of H_37_Rv. (**b**) Densitometry was performed, and the ratios of phosphorylated to total protein expression were expressed as arbitrary units. *P < .05, ** *P* <0.005; *** *P* <0.005 by the Mann–Whitney U

**Fig. 3:**
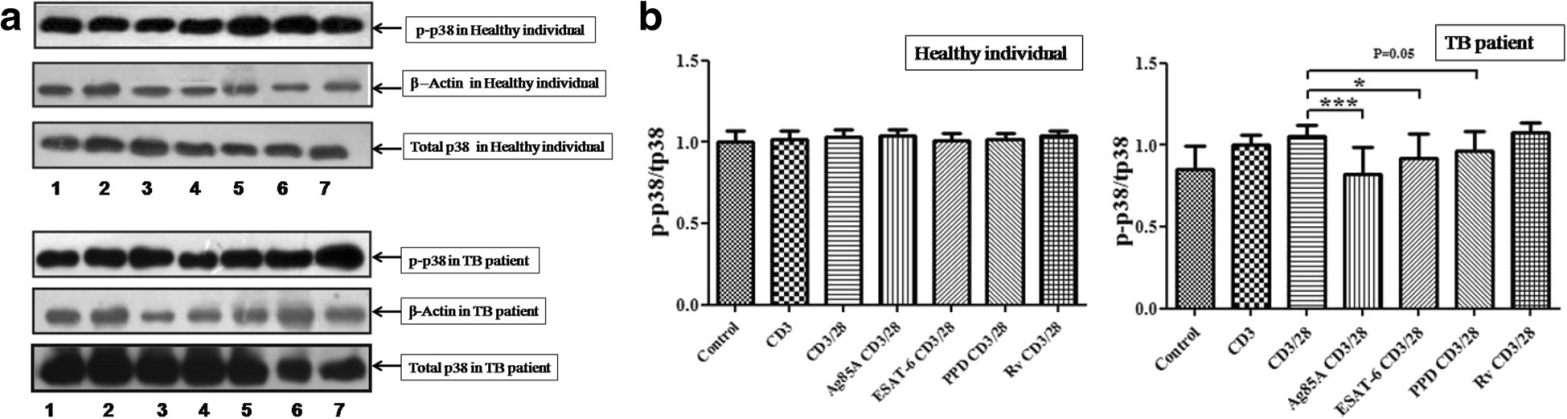
*M. tuberculosis* antigens modulate TCR- and TCR/CD28-induced p38 phosphorylation in PPD+ve healthy individuals and pulmonary TB patients. (**a**) Peripheral blood mononuclear cells (PBMCs) from six PPD + ve healthy donors and 12 pulmonary TB patients were stimulated with *M. tuberculosis* antigens for 4 h. Cells were then activated with anti CD3 and anti CD28 antibodies (as mentioned in methodology). Phosphorylated p38, total p-38 (p- p38 and tp38, respectively) and β- Actin expression were determined by western blot. One representative blot of each study case is shown where Lane 1- Control, Lane 2- anti CD3 stimulated, Lane 3- anti CD3 + anti CD28 stimulated, Lane 4- anti CD3 + anti CD28 stimulated in presence of Ag85A, Lane 5- anti CD3 + anti CD28 stimulated in presence of ESAT-6, Lane 6- anti CD3 + anti CD28 stimulated in presence of PPD and Lane 7-anti CD3 + anti CD28 stimulated in presence of H_37_Rv. (**b**) Densitometry was performed, and the ratios of phosphorylated to total protein expression were expressed as arbitrary units. *P < .05, ** *P* <0.005; *** *P* <0.005 by the Mann–Whitney U test

To determine whether *M. tuberculosis* antigens modulate TCR and TCR/CD28 induced MAPK activation, we measured phosphorylation of ERK ½ and p38 by Western blot. Phosphorylation of MAPKs was studied in Ag85A, ESAT-6, PPD and H_37_Rv stimulated cells of PPD+ve healthy individuals and pulmonary TB patients. Although there was notable individual variation, TCR/CD28 induced phosphorylation of ERK1/2 was observed to be significantly reduced by ESAT-6 in healthy individuals, while H_37_Rv significantly increased phosphorylation of ERK ½. Ag85A had no significant effect on activation of ERK ½, though PPD increased phosphorylaion but not significantly. However, in pulmonary TB patients significant inhibition of TCR/CD28 triggered ERK1/2 phosphorylation by Ag85A and ESAT-6 was observed. PPD had no effect on phosphorylation of ERK ½ and H_37_Rv decreased but not significantly (Fig. 2a and b). The phosphorylation of p38 in TCR/CD28 induced cells was also analysed after stimulation with *M. tuberculosis* antigens. No effect of Ag85A, ESAT-6, PPD and H_37_Rv on p38 phosphorylation in PPD+ve healthy individuals was noted. On the other hand Ag85A and ESAT-6 significantly curtailed phosphorylation of p38 in pulmonary TB patients (Fig. 3a and b).

### *M. tuberculosis* antigens differentially modulate TCR/CD28-induced binding of NFκB and NFAT

**Fig. 4:**
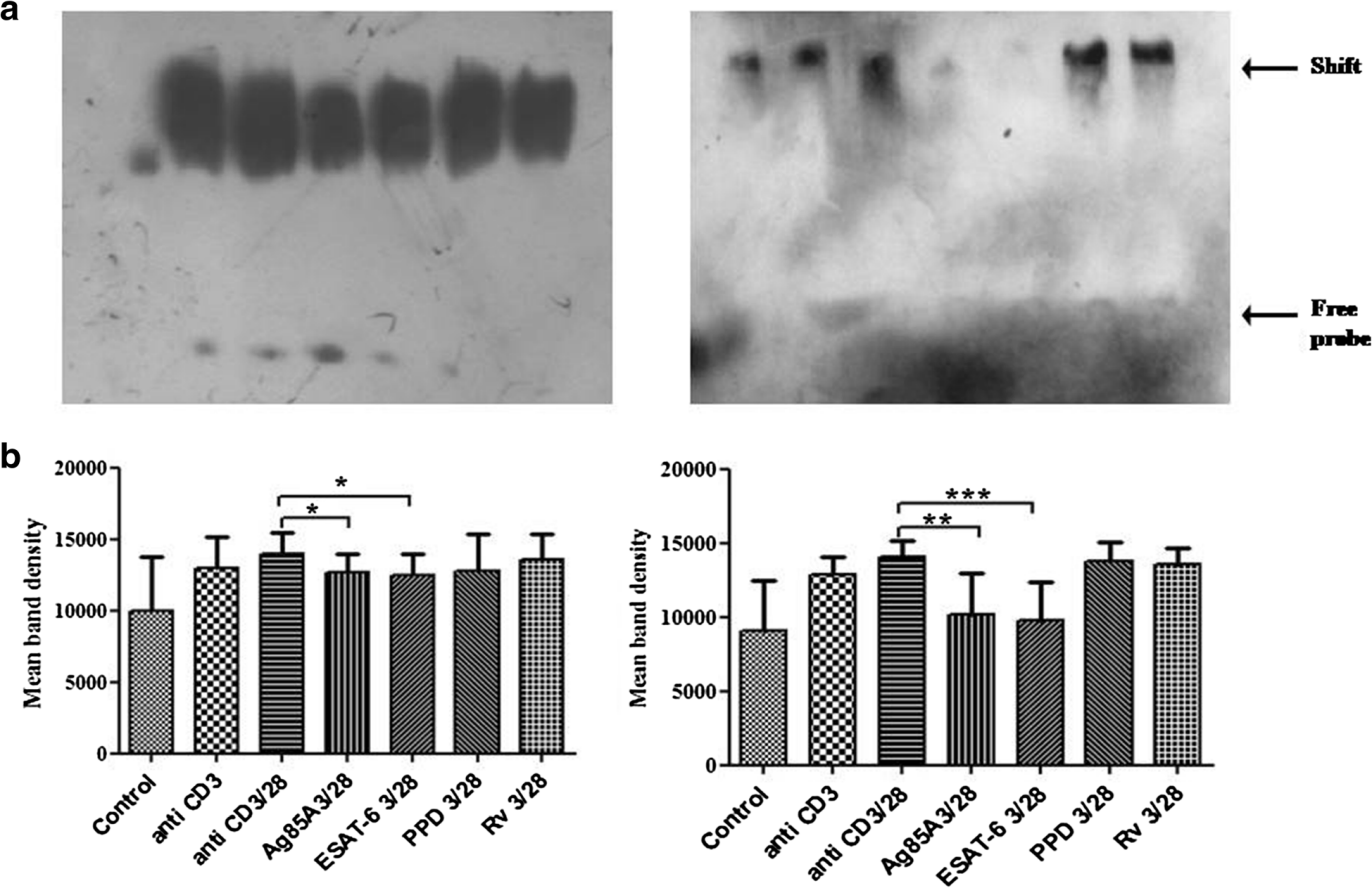
*M. tuberculosis* antigens modulate TCR- and TCR/CD28-induced DNA binding affinity of NFκB to the promoter of IL-2 cytokine in PPD+ve healthy individuals and pulmonary TB patients. (**a**) Peripheral blood mononuclear cells (PBMCs) from 11 PPD+ve healthy donors and 10 pulmonary TB patients were stimulated with *M. tuberculosis* antigens for 4 h. Cells were then activated with anti CD3 and anti CD28 antibodies (as mentioned in methodology). Nuclear lysates were prepared and binding affinity of NFκB was determined by EMSA. One representative blot of each study case is shown where Lane 1- Control, Lane 2- anti CD3 stimulated, Lane 3- anti CD3 + anti CD28 stimulated, Lane 4- anti CD3 + anti CD28 stimulated in presence of Ag85A, Lane 5- anti CD3 + anti CD28 stimulated in presence of ESAT-6, Lane 6- anti CD3 + anti CD28 stimulated in presence of PPD and Lane 7-anti CD3 + anti CD28 stimulated in presence of H_37_Rv. (**b**) Densitometry was performed, Histograms represent mean band intensities. *P < .05, ** *P* <0.005; *** *P* <0.005 by the Mann–Whitney U test

**Fig. 5:**
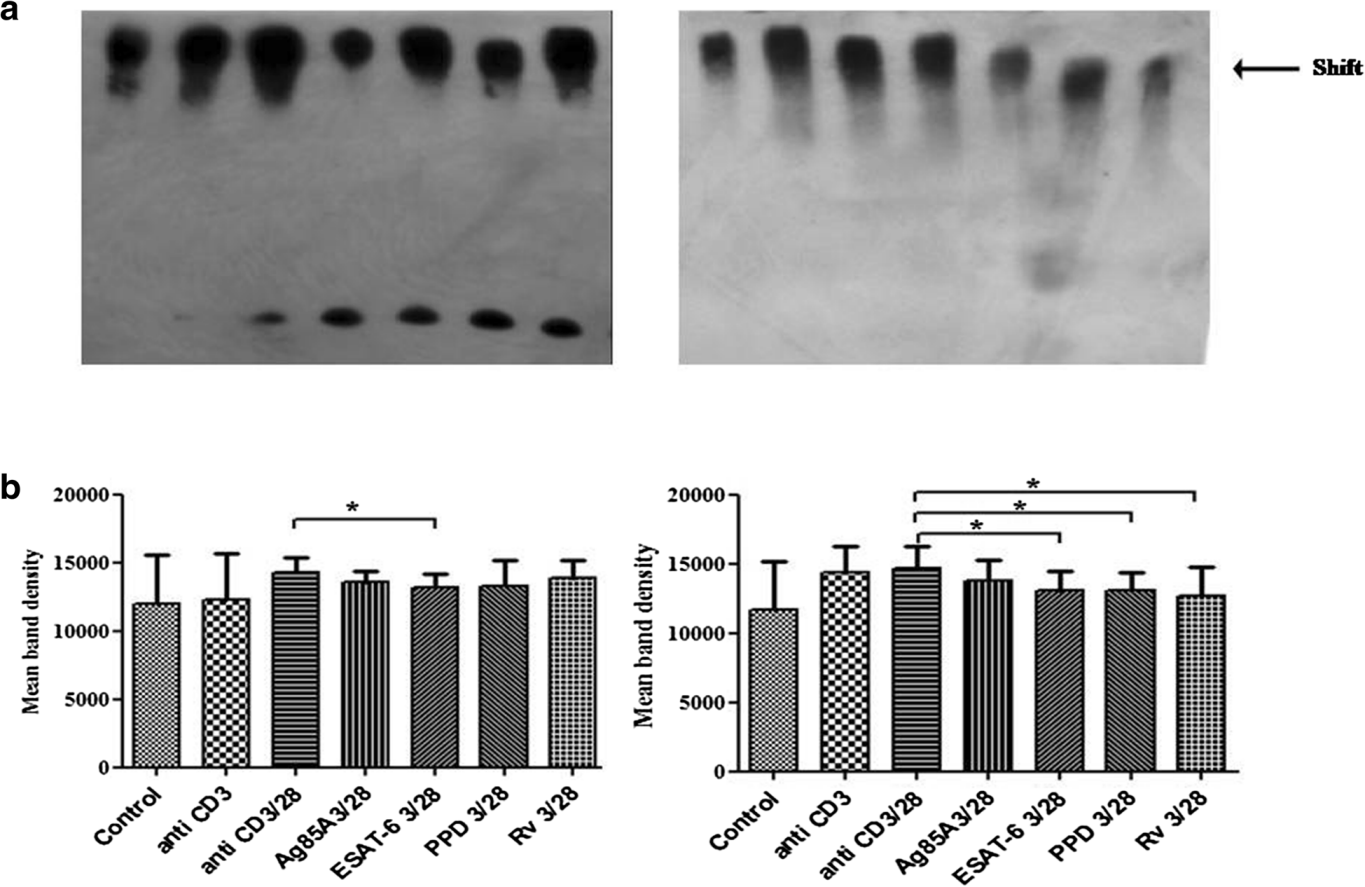
*M. tuberculosis* antigens modulate TCR- and TCR/CD28-induced DNA binding of NFAT to the promoter of IL-2 cytokine in PPD+ve healthy individuals and pulmonary TB patients. (**a**) Peripheral blood mononuclear cells (PBMCs) from 11 PPD+ve healthy donors and 10 pulmonary TB patients were stimulated with *M. tuberculosis* antigens for 4 h. Cells were then activated with anti CD3 and anti CD28 antibodies (as mentioned in methodology). Nuclear lysates were prepared and binding affinity of NFAT was determined by EMSA. One representative example of each study case is shown where Lane 1- Control, Lane 2- anti CD3 stimulated, Lane 3- anti CD3 + anti CD28 stimulated, Lane 4- anti CD3 + anti CD28 stimulated in presence of Ag85A, Lane 5- anti CD3 + anti CD28 stimulated in presence of ESAT-6, Lane 6- anti CD3 + anti CD28 stimulated in presence of PPD and Lane 7-anti CD3 + anti CD28 stimulated in presence of H_37_Rv. (**b**) Densitometry was performed, Histograms represent mean band intensities. *P < .05,** *P* <0.005; *** *P* <0.005 by the Mann–Whitney U test

DNA binding affinity of NFκB and NFAT on IL-2 promoter was also studied on TCR/CD28 activated cells by Electrophoretic mobility shift assay (EMSA). Ag85A, ESAT-6, PPD and H_37_Rv curtailed binding affinity of NFκB in PPD+ve healthy individuals but only ESAT-6 showed statistically significant reduction in binding affinity. In pulmonary TB patients Ag85A and ESAT-6 significantly curtailed the binding affinity of NFκB (Fig. 4a and b). We also noted that Ag85A, ESAT-6, PPD and H_37_Rv curtailed TCR/CD28 induced binding activity of NFAT on IL-2 promoter in PPD+ve healthy individuals and pulmonary TB patients both. ESAT-6 was the only antigen that curtailed the binding activity of NFAT significantly in healthy individuals and patients both, but significant inhibition by PPD and H_37_Rv was observed in patients only, though no significant effect of Ag85A was noted in healthy and patients both (Fig. 5a and b).

## Discussion

The outcome of TB mainly depends on T-cell response, which is altered through multiple, but poorly understood mechanisms. This study was designed to investigate the impact of *M. tuberculosis* antigens on T cell activation and their role in modulation of T-cell physiology in disease, which still needs to be defined. Here, we observed that patients with active pulmonary TB have a significant diminished TCR triggered intracellular calcium mobilization in response to secretory *M. tuberculosis* antigens which is an important regulatory signal-transduction stage of T cell activation. Further, selective modulation in TCR/CD28-induced late signalling events like MAPKs and binding of NFAT and NFκB which is important in the activation of certain T cell genes for cytokine production was also noted in presence of *M. tuberculosis* antigens. The data suggests that these alterations could explain the loss of a protective immune response by reducing production of proinflammatory cytokines IL-2 against *M. tuberculosis* to some extent, and it may also help us to figure out the mechanisms leading to T cell dysfunction in TB.

Optimal T cell activation involves stimulation through the TCR by MHC-peptide complex along with additional signalling through co-stimulatory receptors. The TCR is a complex consisting intracellular signal transducing domains, immunoreceptor tyrosine-based activation motifs (ITAMs). Once phosphorylated, ITAMS become recruitment sites for ZAP-70, activated Zap-70 is then recruited to phosphorylate the multiple transmembrane molecules. These signalling molecules activate multiple secondary signalling pathways including Ca^2+^ signalling, Ras/MAP kinase, and protein kinase C (PKC) pathways that ultimately activate several transcription factors including NFAT, AP-1 and NF-κB that are essential for full IL-2 gene expression and T cell activation [12–14]. We observed that ESAT-6 curtailed TCR triggered calcium mobilization in pulmonary TB patients and PPD+ve healthy individuals both but the inhibition is more in patients as compared to healthy individuals. On the other hand, Ag85A, PPD and H_37_Rv diminished transmembrane calcium mobilization in pulmonary TB patients but enhanced the transmembrane calcium influx in PPD+ve healthy individuals. Present study confirms that mycobaterial antigens: Ag85A, ESAT-6, PPD and H37Rv, altered calcium signalling and consequently might play a critical role in the pathogenesis of the disease. Altered calcium signalling and other signalling events has been noted in leprosy patients and T cell lines and modulation by lipid and soluble fraction of *M. leprae* lysates was shown which could play a major role in the pathogenesis [15–19]. Our results are in close concordance with other reports where PBMCs from patients with leprosy have been reported to have reduced cytosolic [Ca^+2^]i concentrations [16, 17]. In macrophages curtailment of [Ca^+2^]i levels by *M. tuberculosis* has been shown, which could be linked with reduced phagosome-lysosome fusion, thus for increased survival of mycobacteria [20]. Only one report is available on intracellular calcium modulation by H_37_Rv in newly diagnosed and treated TB patients and PPD+ve and PPD-ve healthy controls [21]. In addition, we used PPD and immunodominant *M. tuberculosis* secretory antigens. On the contrary, it has been previously reported that ESAT-6 did not affect phosphorylation of ZAP-70 and intracellular calcium levels but ESAT-6 inhibits T cell immune responses by affecting downstream signalling molecules [7].

The importance of the MAPKs signal transduction pathway in controlling many aspects of immune-mediated inflammatory responses has made it a priority for research, related to many human diseases. Therefore, we further studied downstream signalling events such as activation of MAPKs and nuclear translocation of transcription factors NFκB and NFAT, important events for both cytokine production and cell activation. Interestingly, we observed that ESAT-6 significantly curtailed TCR/CD28 induced phosphorylation of ERK1/2 in healthy individuals and pulmonary TB patients both. We did not observe any effect on p38 phosphorylation in TCR/CD28 induced cells of healthy individual after treatment with *M. tuberculosis* antigen whereas in pulmonary TB patients Ag85A and ESAT-6 significantly curtailed p38 phosphorylation. Palma-Nicholas [22] reported down-modulation of MAPK–ERK1/2 pathway in total spleen cells from naive BALB/c mice by the cell-surface lipid di-O-acyl-trehalose (DAT), which further leads to lowering of Th-1 type cytokine production. As per our knowledge ours is the first study where differential modulation of TCR or TCR/CD28 induced downstream signalling events like MAPKs in the PBMCs of pulmonary TB patients has been observed. The finding suggests modulation of ERK1/2 and p38 both or majorly p38 in T cells by *M. tuberculosis* that could lead to T cell dysfunction or low Th1 cytokine production. Inhibition of IFN-γ by ESAT-6 in human T cells through p38 MAPK has been reported earlier [11], however, our observation of inhibition of p38 by Ag85A in TB patients is a novel information and confirms the role of p38 in the T cell dysfunction. Similar to our findings differential modulation of downstream signalling events like MAPKs by *M. leprae* antigens stimulated T cells from leprosy patients have been reported earlier [16, 17]. Further, diminished and differential phosphorylation of MAPKs by *M. leprae* antigens in T cell lines after stimulation with anti CD3 or anti CD3+CD28 has also been reported [18, 19]. Effect of secretory protein ESAT-6 of *M. tuberculosis* in the modulation of macrophage signalling pathways particularly ERK1/2 MAPK pathway has been shown in one study [23].

Further, binding affinity of NFκB and NFAT of antigen treated cells in pulmonary TB patients and in PPD+ve healthy individuals was also assessed by EMSA. We observed that ESAT-6 significantly reduced the binding of NFκB in healthy individuals and pulmonary TB patients. We also noticed curtailment of binding activity of NFAT in anti CD3+CD28 stimulated cells in pulmonary TB patients after treatment with ESAT-6, PPD and H_37_Rv. ESAT-6 induced cells were showing significant inhibition of NFAT binding in pulmonary TB patients and healthy individuals both. Our findings of reduced NFAT binding in ESAT-6 stimulated cells in healthy individuals and TB patients could be correlated with the reduced Ca^2+^ mobilization of ESAT −6 stimulated cells in healthy and patients both. Calcium is an essential element in many T-cell responses, including a pathway that leads to the movement of a major transcription factor, NFAT, from the cytoplasm into the nucleus where NFAT supports the transcription of genes required for the expression of the T cell growth-promoting cytokines IL-2 [13]. No study on the expression of NFkB and NFAT in activated T cells in TB patients is available till date, however, Zea et al. [24] have noticed reduced expression of p65 of NFkB in T cells of pulmonary TB patients. Tchou- Wong et al. [25] revealed specific binding of nuclear protein to the NFkB site upon induction with *M. tuberculosis* in human monocytic leukemic cell line THP-1. Impaired nuclear translocation of NFκB and NFAT was also noticed in leprosy patients [15–17].

While inhibition of T-cell activation is a novel observation for *M. tuberculosis*, it has been shown to occur with other pathogens. These include bacteria, viruses, and protozoan parasites. Bacterial toxins, including those from *Helicobacter pylori* and *Bacillus anthracis*, can suppress TCR signalling by blocking calcium influx and MAPKs, respectively [26–28]. *Salmonella enterica* may also express a protein(s) that can down-modulate TCR expression [29].

Our study indicates that *M. tuberculosis* has several ways to destabilize the host’s T-cell response besides what is already established. Although previous studies of cell signalling pathways in TB have contributed upto some extent to our knowledge of their role in host protective immune responses, a number of critical questions are not well understood. Research into the development of TB vaccines and immunodiagnostics has focused on the proteins released by *M. tuberculosis*, because these antigens are thought to induce protective cell mediated immunity and immune responses of diagnostic value. Ag85A and ESAT-6 are widely studied for their potential to trigger effective host immune responses against TB, but very little is known regarding their role in the T cells signalling mechanisms underlying proinflammatory cytokine secretion by T cells. Our data demonstrate that the stimulation of human peripheral blood cells *in vitro* with the Ag85A and ESAT-6 antigens of *M. tuberculosis* markedly down modulated TCR activation by reducing activation of T cells by affecting upstream as well as downstream TCR signalling events. We suggest that these mycobacterial antigens can affect activation of intracellular T cell signalling pathways, such as calcium mobilization, MAPKs that are required for production of IL-2. Further work is required to delineate the molecular mechanisms underlying these effects, to better understand interactions between *M. tuberculosis* and human immune system and to facilitate development of ESAT-6 and Ag85A-based vaccines.

## Conclusion

This study has shown that a significant number of the patients with pulmonary TB had alterations in the expression of several T cell signal-transduction proteins, alterations that were similar to those reported earlier in patients with leprosy. The similarity of T cell signal-transduction alterations in diseases with different pathophysiological characteristics suggests the possibility that a common mechanism causes such changes. Follow-up studies are needed to determine whether such alterations revert after successful treatment and also whether these can be modulated by immunotherapy. It would also be of interest to know variations in different forms of tuberculosis and determine relationship with activation of different cytokines. These observations of molecular and functional characteristics in TB may provide new tools to analyse and monitor patients, to reveal how these characteristics affect the development of immune dysfunction and to study new pathways to block suppressor mechanisms. This endeavour will enhance our knowledge of disease pathogenesis, contributing to a better understanding of the immune response *to M. tuberculosis*.

## Methods

### Study subjects

During the study period of two years 2008–2010, we recruited 30 human immunodeficiency virus (HIV) - seronegative patients with TB diagnosed at State Tuberculosis Demonstration Centre (STDC), Agra. The diagnosis of TB was established based on clinical and radiological data together with the positive identification of acid-fast bacilli in sputum. Fresh active pulmonary TB cases were included in the study. Patients recruited before initiation of Anti TB Treatment (ATT of 2 months intensive phase of Isoniazid, Rifampicin, Pyrazinamide and Ethambutol and 4 months continual phase of Isoniazid and Rifampicin) and receiving ATT for less than a month were considered as fresh case as per RNTCP guidelines (Revised National Tuberculosis Control Program) [30]. We also included 27 healthy adults with no history of tuberculosis who were working in the National JALMA Institute for leprosy and other Mycobacterial Diseases, Agra. They showed absence of any clinical disease and were positive for the purified protein derivative (PPD). The study was carried after taking the approval from Institutional human Ethics Committee set up as per the guidelines of Indian Council of Medical Research, New Delhi, India. Twenty ml of blood sample was collected from all participants after taking informed written consent.

### Antigen

Lyophilized *M. tuberculosis* antigens (Ag85A, ESAT-6 and whole cell lysate of H_37_Rv), used in this study were provided by J. Belisle (Colorado State University, Denver, CO, USA) through a TB Research Materials and Vaccine Testing Contract (NIH Contract HHSN266200400091C/ADB Contract NOI-AI-40091). PPD (RT-49) was procured from Statens Serum Institute, Copenhagen, Denmark. PPD and whole cell lysate of H_37_Rv were used as positive antigen control. PHA was procured from (Sigma, St. Loius, MO, USA) and used as positive control only in LTT experiments. All antigens were dissolved in filtered Phosphate buffered saline (PBS) pH 7.4 to make 1 mg/ml concentration.

### Cell preparation and reagents

Study was performed on peripheral blood mononuclear cells (PBMCs) isolated from buffy coat obtained from blood of pulmonary TB patients and PPD+ve healthy individuals, by density gradient centrifugation over Ficoll- HyPaque (Sigma, St. Loius, MO, USA). Cells were maintained in RPMI-1640 medium supplemented with 5 % heat inactivated FBS (Hyclone, Utah, USA) with 2 mM L-glutamine and 1× antibiotic–antimycotic cocktail (Sigma, St. Loius, MO, USA). Cultures were maintained with 5 % CO_2_ at 37 °C in a humidified chamber. Experiments were performed with cell viability >95 % as determined by Trypan blue exclusion test.

Mouse IgG anti-human pure CD3 (clone UCHT1), Ionomycin, goat anti-mouse-IgG (GAM), phenylmethylsulphonyl fluoride (PMSF), sodium orthovanadate, anti-protease cocktail, Bradford reagent and Nuclear protein extraction kit were purchased from Sigma, St. Louis, MO, USA. Fura-2/AM was procured from Calbiochem, La Jolla, USA. Anti-human CD3 (clone HIT3-α), anti-human CD28 (clone CD28.2) were procured from BD Biosciences, CA, USA. Phospho-ERK1/2 and phospho-p38MAPK antibodies were procured from Cell Signalling Technology, MA, USA. Anti-ERK −2, anti p38, β-actin and peroxidase-conjugated goat anti-mouse/goat anti-rabbit secondary antibodies were purchased from Santacruz Technologies, CA, USA. ECL reagents were procured from Millipore, MA, USA.

### Quantification of transmembrane Ca^2+^ mobilisation

PBMCs (5 × 10^6^/ml) from PPD+ve healthy individuals (*N* = 13) and pulmonary TB patients (*N* = 5) were stimulated with appropriate doses of *M. tuberculosis* antigens: 5 μg/ml Ag85A, 10 μg/ml of ESAT-6, 5 μg/ml PPD and 5 μg/ml of H_37_Rv, for 4 h in 37 °C CO_2_ incubator. After incubation cells were washed with PBS, pH 7.4. Cells were incubated with Fura-2/AM at 1 μM concentration for 30 min at 37 °C in loading buffer [(in mM): NaCl, 110; KCl, 5.4;NaHCO_3_, 25; MgCl_2_, 0.8;KH_2_PO4, 0.4; HEPES, 20;Na_2_HPO4, 0.33; CaCl_2_, 1.2. pH 7.4]. After loading, cells were washed three times (500 × g for 5 min) and remained suspended in the identical buffer. [Ca^2+^]i was measured as reported elsewhere [31]. Fluorescence intensities were measured in ratio mode using Varian ECLIPSE spectrofluorometer equipped with Fast filter accessory (Varian Incorporation, St. Helens, Australia) at 340 and 380 nm (excitation filters) and 510 nm (emission filter). Cells were stirred continuously throughout the experiment. For anti CD3 stimulated calcium studies: after stabilization of basal levels of cytosolic calcium, 10 μg/ml of pure Anti-CD3 (Clone UCHT1) was added to cuvette. For measurement of Fmax, Ionomycin 5 μM was added to cuvette and for F min MnCl_2_ 2 mM was added.

The intracellular concentrations of free Ca^2+^ [Ca^2+^]i, were calculated by using the following equation: [Ca^2+^]i = Kd × (R − Rmin)/(Rmax − R) × (Sf2/Sb2). A value of 224nM for Kd was added into the calculations. Rmax and Rmin values were obtained by addition of ionomycin (5 μM) and MnCl_2_ (2 mM), respectively. All experiments were performed at 37 °C.

### Treatment and activation of cells

PBMCs (5 × 10^6^/ml) were stimulated with appropriate doses of *M. tuberculosis* antigens: 5 μg/ml Ag85A, 10 μg/ml of ESAT-6, 5 μg/ml PPD and 5 μg/ml of H_37_Rv, for four hours in 37 °C CO_2_ incubator, then stimulated with or without anti-CD3 antibody (10 μg/ml) alone for 15 min and then with anti-CD28 antibody (5 μg/ml) for 10 min at 4 °C. Further, cross linking was done using GAM-IgG (5 μg/ml) for 15 min at 37 °C.

### Western blot analysis of MAPKs activation

Antigen stimulated and anti CD3 and CD28 activated cells from PPD+ve healthy individuals (*N* = 6) and pulmonary TB patients (*N* = 12) were treated with chilled PBS and then lysed with 50 μl of buffer (HEPES 20 mM, pH 7.3; EDTA1mM; EGTA 1 mM; NaCl 0.15 mM; Triton X-100 1 %; glycerol 10 %; PMSF 1 mM; sodium orthovanadate 2 mM; anti-protease cocktail). After centrifugation (13,000 × g for 5 min), cell lysates were used immediately or stored at −80 °C. The protein contents were determined with Bradford reagent. Denatured proteins (30 μg) were separated by SDS-PAGE (10 %) and transferred to polyvinylidine difluoride (PVDF) membranes. Immunodetection of phosphorylated forms of ERK1/2 and p38MAPK was performed using 2 μg/ml of phospho-specific antibodies in TBS with 2.5 % BSA with overnight incubation at 4 °C. After washing with TBST (TBS with 0.05 % Tween-20), PVDF membranes were treated with peroxidase-conjugated goat anti-mouse/anti-rabbit secondary antibodies and peroxidase activity was detected with ECL reagents. Equal loading of the proteins was confirmed after striping the blot and reprobing for total forms of ERK-2/β-actin. Densitometric analysis of bands was performed using Quantity OneTM software (Bio-Rad, Hercules, USA).

### Purification of nuclear proteins for EMSA

Nuclear extracts were prepared as described in Cell Lytic NuCLEAR Extraction kit. Briefly, antigens pre treated and anti CD3 and anti CD3/CD28 activated cells from PPD+ve healthy individuals (*N* = 11) and pulmonary TB patients (*N* = 10) were washed with ice-cold PBS and then lysed by incubation in 100 μl of 1X lysis buffer (50 mM Tris HCl, pH 7.5, with 10 mM MgCl_2_, 15 mM MgCl_2_, 15 mM CaCl_2_, and 1.5 M Sucrose, 1 μl of 0.1 M DTT and 1 μl of protease inhibitor cocktail on ice for 15 min. Nuclei were pelleted by centrifugation at 10,000xg for 30 s at 4 °C, the supernatant was saved as the cytoplasmic extract, and the nuclear pellet was resuspended in 100 μl of extraction buffer (20 mM HEPES, pH 7.9, with 1.5 mM MgCl_2_, 0.42 M NaCl, 0.2 mM EDTA, 25 %(*v/v*) Glycerol, 1 μl of 0.1 M DTT and 1 μl of protease inhibitor cocktail). The tubes were mounted on a vortex mixture and agitated at medium to high speed for 15–30 min. Tubes were then centrifuged at 20,000 x g for 5 min, and the supernatant was nuclear extract which is then transferred in a fresh chilled tube and were stored at −80 °C.

### Labelling of probes for EMSA

Synthetic complementary oligonucleotides were 3′- biotinylated using the biotin 3′-end DNA labeling kit (Pierce- Endogen, Illinois, USA) according to the manufacturer’s instructions and annealed for two hours at room temperature. The sequence of the oligonucleotide used for NFAT was 5′-GATCTTTACATTGGAAAATTTTAT- 3′ [32] and for NF-κB, 5′-AGCTTAGAGGGGACTTTCCGAGAGGA-3′ [33].

### Analysis of EMSA

Binding reactions were carried out for 20 min at room temperature in the presence of 50 ng/ml polyI:C, 0.05 % NP-40, 5 mM MgCl_2_, 10 mM EDTA, and 2.5 % glycerol in 1× binding buffer (LightShiftTM chemiluminescent EMSA kit, Pierce-Endogen, Illinois, USA) using 20 fmol of biotin-end-labeled target DNA with 4 μg of nuclear extract. Assays were loaded onto native 4 % polyacrylamide gels pre-electrophoresed for 60 min in 0.5× Tris-borate/EDTA and then gels were electrophoresed at 100 V before transferring to positively charged nylon membrane (Immobilion-NY, Millipore, MA, USA) in 0.5× Tris-borate/EDTA at 100 V for 30 min. Transferred DNA was UV cross-linked to the membrane at 120 mJ/cm^2^ and detected using horseradish peroxidase-conjugated streptavidin as per manufacturer’s instructions. Peroxidase activity was detected with Enhanced chemiluminiscence (ECL) reagents. Densitometric analysis of bands was performed using Quantity OneTM software (Bio-Rad, Hercules, USA).

### Statistical analysis

We used GraphPad Prism v5.0 (La Jolla, USA) for statistical analysis. The Mann–Whitney *U* test was used to analyze differences between unpaired samples. P values of <0.05 were considered statistically significant.

## Additional file

Additional file 1: Figure S1. (A-C) Lymphocyte transformation test for dose optimisation of Ag85A, ESAT-6 and H_37_Rv by using PBMCs of healthy individuals (*N* = 10) and pulmonary TB (*N* = 10) patients using H^3^-thymidine uptake assay. PHA and PPD were used as positive control and wells with unstimulated cells were taken as control. Lymphoproliferative responses of healthy individuals and pulmaonary TB patients were calculated using different concentration (2.5, 5, 7.5, 10, 15, μg/ml) of antigens. Bar diagram showing mean ± SEM of stimulation indices (S.I) of stimulated PBMC’s with different doses of PHA, PPD, A85A, ESAT-6 and H_37_Rv. S.I was calculated according to the formula: $\mathrm{S}\mathrm{I}=\frac{\mathrm{Mean}\ \mathrm{counts}\ \mathrm{per}\ \mathrm{minute}\ \mathrm{of}\ \mathrm{experimental}\ \mathrm{wells}}{\mathrm{Mean}\ \mathrm{counts}\ \mathrm{per}\ \mathrm{minute}\ \mathrm{of}\ \mathrm{control}\ \mathrm{wells}}$ (ZIP 160 kb)

## Abbreviations

IFN-γ: Interferon gamma
IL-2: Interleukin 2
ESAT-6: Early secretory antigenic target −6
PPD: Purified protein derivative
[Ca+2]i: Free intracellular calcium concentrations
