# Supplementary figures and images for: Mycobacterium tuberculosis secretory proteins downregulate T cell activation by interfering with proximal and downstream T cell signalling events
Source: BMC Immunol. 2015 Nov 9;16:67. doi: 10.1186/s12865-015-0128-6 (PMC4640201; doi:10.1186/s12865-015-0128-6)

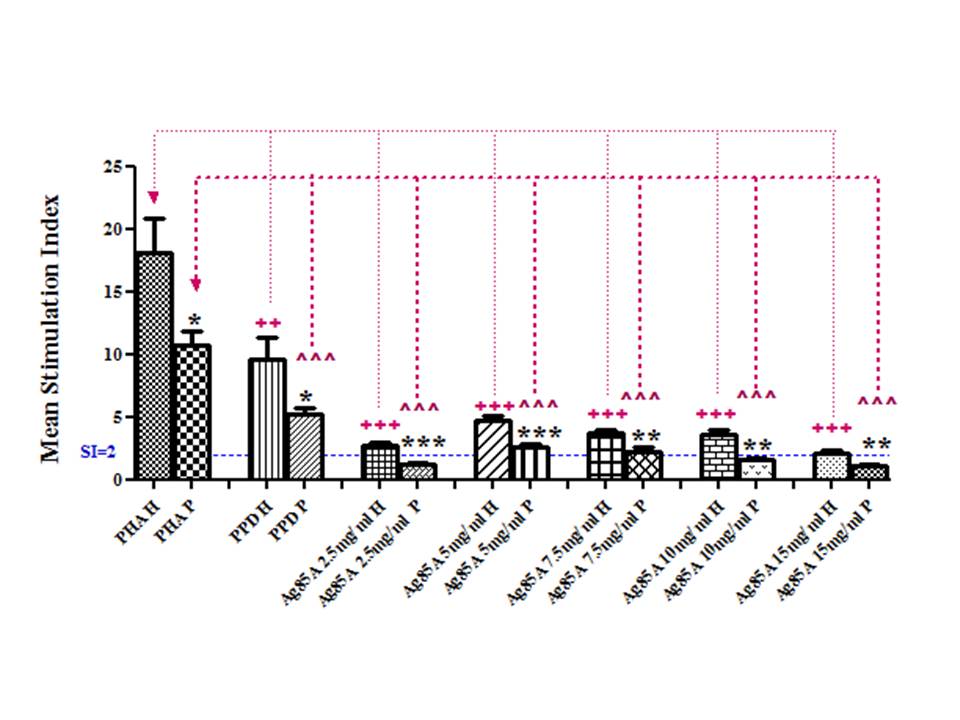

Supplement: Additional file 1: Figure S1. — (A-C) Lymphocyte transformation test for dose optimisation of Ag85A, ESAT-6 and H37Rv by using PBMCs of healthy individuals (N = 10) and pulmonary TB (N = 10) patients using H3-thymidine uptake assay. PHA and PPD were used as positive control and wells with unstimulated cells were taken as control. Lymphoproliferative responses of healthy individuals and pulmaonary TB patients were calculated using different concentration (2.5, 5, 7.5, 10, 15, μg/ml) of antigens. Bar diagram showing mean ± SEM of stimulation indices (S.I) of stimulated PBMC’s with different doses of PHA, PPD, A85A, ESAT-6 and H37Rv. S.I was calculated according to the formula: $\mathrm{S}\mathrm{I}=\frac{\mathrm{Mean}\ \mathrm{counts}\ \mathrm{per}\ \mathrm{minute}\ \mathrm{of}\ \mathrm{experimental}\ \mathrm{wells}}{\mathrm{Mean}\ \mathrm{counts}\ \mathrm{per}\ \mathrm{minute}\ \mathrm{of}\ \mathrm{control}\ \mathrm{wells}}$SI=MeancountsperminuteofexperimentalwellsMeancountsperminuteofcontrolwells (ZIP 160 kb) [file 12865_2015_128_MOESM1_ESM.zip › Additional file 1/Additional file 1 Figure S1) A.JPG]

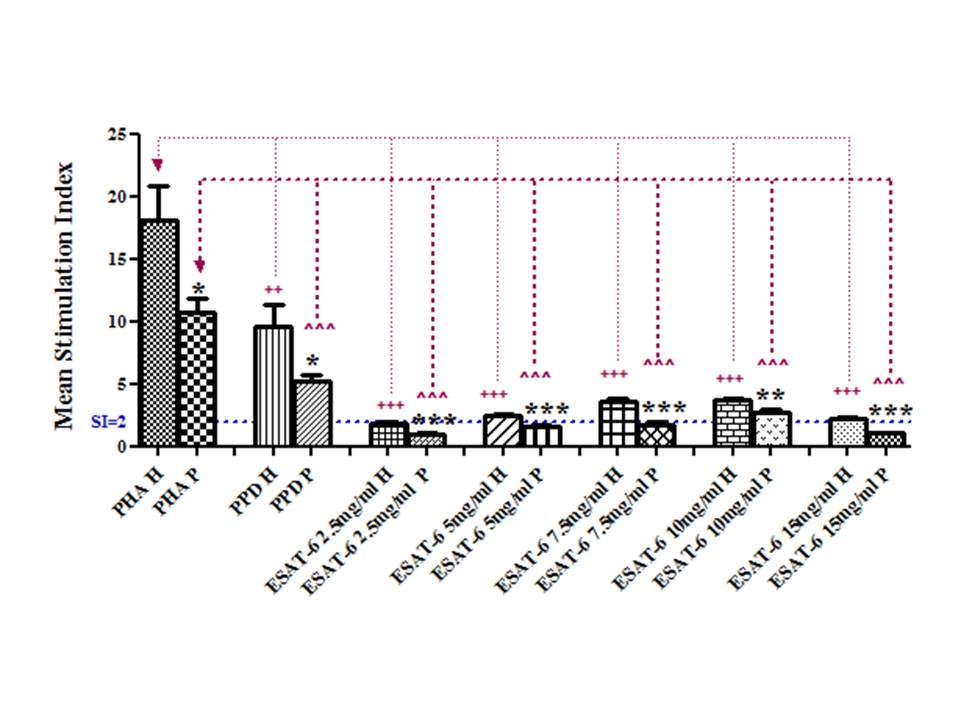

Supplement: Additional file 1: Figure S1. — (A-C) Lymphocyte transformation test for dose optimisation of Ag85A, ESAT-6 and H37Rv by using PBMCs of healthy individuals (N = 10) and pulmonary TB (N = 10) patients using H3-thymidine uptake assay. PHA and PPD were used as positive control and wells with unstimulated cells were taken as control. Lymphoproliferative responses of healthy individuals and pulmaonary TB patients were calculated using different concentration (2.5, 5, 7.5, 10, 15, μg/ml) of antigens. Bar diagram showing mean ± SEM of stimulation indices (S.I) of stimulated PBMC’s with different doses of PHA, PPD, A85A, ESAT-6 and H37Rv. S.I was calculated according to the formula: $\mathrm{S}\mathrm{I}=\frac{\mathrm{Mean}\ \mathrm{counts}\ \mathrm{per}\ \mathrm{minute}\ \mathrm{of}\ \mathrm{experimental}\ \mathrm{wells}}{\mathrm{Mean}\ \mathrm{counts}\ \mathrm{per}\ \mathrm{minute}\ \mathrm{of}\ \mathrm{control}\ \mathrm{wells}}$SI=MeancountsperminuteofexperimentalwellsMeancountsperminuteofcontrolwells (ZIP 160 kb) [file 12865_2015_128_MOESM1_ESM.zip › Additional file 1/Additional file 1 Figure S1) B.JPG]

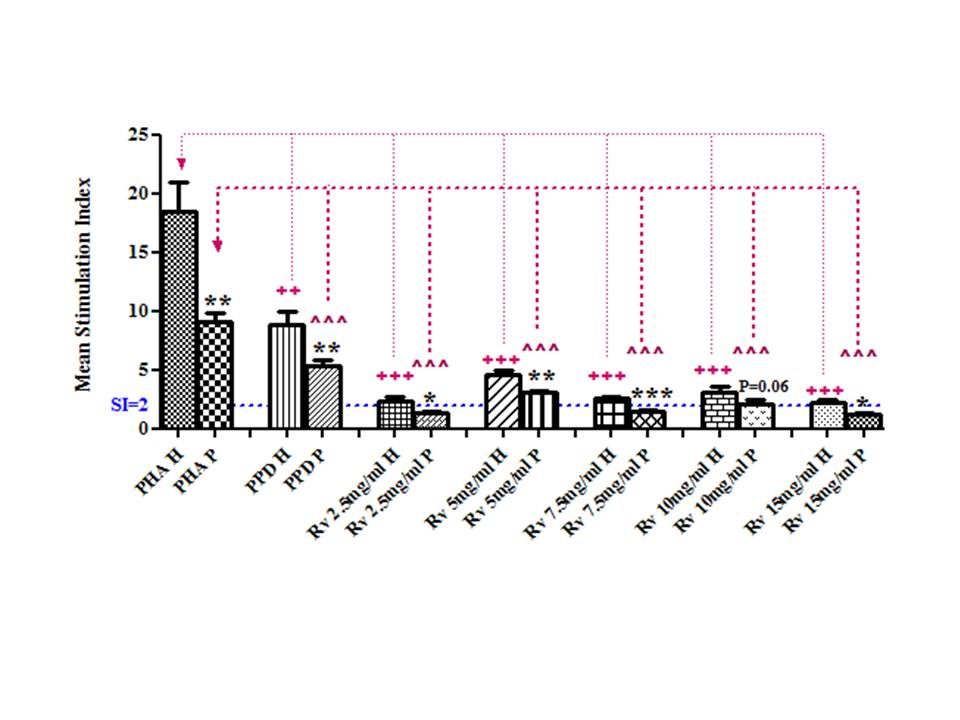

Supplement: Additional file 1: Figure S1. — (A-C) Lymphocyte transformation test for dose optimisation of Ag85A, ESAT-6 and H37Rv by using PBMCs of healthy individuals (N = 10) and pulmonary TB (N = 10) patients using H3-thymidine uptake assay. PHA and PPD were used as positive control and wells with unstimulated cells were taken as control. Lymphoproliferative responses of healthy individuals and pulmaonary TB patients were calculated using different concentration (2.5, 5, 7.5, 10, 15, μg/ml) of antigens. Bar diagram showing mean ± SEM of stimulation indices (S.I) of stimulated PBMC’s with different doses of PHA, PPD, A85A, ESAT-6 and H37Rv. S.I was calculated according to the formula: $\mathrm{S}\mathrm{I}=\frac{\mathrm{Mean}\ \mathrm{counts}\ \mathrm{per}\ \mathrm{minute}\ \mathrm{of}\ \mathrm{experimental}\ \mathrm{wells}}{\mathrm{Mean}\ \mathrm{counts}\ \mathrm{per}\ \mathrm{minute}\ \mathrm{of}\ \mathrm{control}\ \mathrm{wells}}$SI=MeancountsperminuteofexperimentalwellsMeancountsperminuteofcontrolwells (ZIP 160 kb) [file 12865_2015_128_MOESM1_ESM.zip › Additional file 1/Additional file 1 Figure S1) C.JPG]
